# Persistent Abnormalities of Fatty Acids Profile in Children With Idiopathic Nephrotic Syndrome in Stable Remission

**DOI:** 10.3389/fped.2020.633470

**Published:** 2021-01-27

**Authors:** Stefano Turolo, Alberto C. Edefonti, William Morello, Marie-Louise Syren, Valentina De Cosmi, Luciana Ghio, Chiara Tamburello, Erika A. Demarco, Alfredo Berrettini, Gianantonio Manzoni, Carlo Agostoni, Giovanni Montini

**Affiliations:** ^1^Pediatric Nephrology, Dialysis and Transplant Unit, Fondazione IRCCS Ca' Granda Ospedale Maggiore Policlinico, Milan, Italy; ^2^Department of Clinical Sciences and Community Health, University of Milan, Milan, Italy; ^3^Pediatric Intermediate Care Unit, Fondazione IRCCS Ca' Granda Ospedale Maggiore Policlinico, Milan, Italy; ^4^Pediatric Urology Unit, Fondazione IRCCS Ca' Granda Ospedale Maggiore Policlinico, Milan, Italy

**Keywords:** idiopathic nephrotic syndrome (INS), fatty acids (FA), arachidonic acids, stable remission, omega-6 (ω-6) PUFA

## Abstract

Steroid-sensitive nephrotic syndrome is an immunological disorder mediated by still poorly defined circulating factor(s) that target the podocyte and damage the filtration barrier. Fatty acids (FA) have several biological roles and, in particular, are strictly involved in cell to cell communication, inflammatory processes and regulation of lymphocyte pools. Studies of FAs during INS have been mainly focused on biochemical changes during the phase of proteinuria; while no information is available about FA profile in patients with idiopathic nephrotic syndrome (INS) on stable remission. Aim of this study is to assess differences in blood FA profile between pediatric patients with INS during the phase of stable remission. Blood fatty acid profile of 47 pediatric patients on stable remission and 47 matched healthy controls were evaluated with gas chromatography. Patients with INS on stable remission had significantly higher levels of PUFA and omega-6 than controls (40.17 vs. 37.91% and 36.95 vs. 34.79%), lower levels of SFA and MUFA. Considering the single fatty acids, levels of omega-6 18:2n6 linoleic acid and omega-6 20:4n6 arachidonic acid were significantly higher in patients with INS than in controls (23.01 vs. 21.55%, *p*-value 0.003 and 10.37 vs. 9.65%, *p*-value 0.01). Moreover, patients with INS showed lower levels of SFA 14:0 (0.74 vs. 0.92%) and 18:0 (10.74 vs. 11.74%) and MUFA 18:1n9 oleic acid (18.50 vs. 19.83%). To the best of our knowledge this is the first study assessing FAs profile in children with INS in stable remission. In a population of 47 patients, we were able to demonstrate a higher blood level of linoleic and arachidonic acid, and consequently of omega-6 and PUFA, compared to controls. Persistently higher than normal levels of either linoleic or arachidonic acid, could be viewed as candidate biomarker for a state of risk of relapse in children with idiopathic nephrotic syndrome.

## Introduction

Childhood idiopathic nephrotic syndrome (INS) is characterized by the presence of heavy proteinuria (≥50 mg/kg/day), serum albumin <25 g/L, and edema (1–5). Children who respond to corticosteroids are defined as steroid-sensitive and have a favorable long-term prognosis; however, among them, those with relapsing episodes of proteinuria may require additional immunosuppressant therapy, which includes calcineurin inhibitors (6), mycophenolate mofetil, and more recently rituximab (7).

Steroid-sensitive nephrotic syndrome is an immunological disorder (1), mediated by still poorly defined circulating factor(s) that target the podocyte and damage the filtration barrier, causing proteinuria. There are some hypotheses on the genesis of the various proposed serum circulating factor(s) (8): T cells, B cells, and podocytes have been indicated as putative secretory cells (9), regulating B-lymphocytes homeostasis (10). A role of inflammation in childhood INS, driven by the activity of IL-17A (11) is supported by the fact that renal epithelial cells synthesize C-Reactive Protein (CRP) in a proteinuria independent manner (12); moreover, an increased expression of Tumor Necrosis Factor-α (TNF-α) in CD4-lymphocytes, with a concomitant decrease of expression of Interferon gamma (IFN-γ) in CD8-lymphocytes, regardless the proteinuria level (13), and an expression dysfunction of Toll Like Receptor-3 (TLR-3) (14) were observed in patients with INS.

Fatty acid metabolism is an emerging field of research in healthcare, not only under the nutritional aspect, but also within a more comprehensive and holistic approach, aimed at finding possible biomarkers for the cardiovascular risk (15, 16). Fatty acids have several biological roles and, in particular, are strictly involved in cell to cell communication, inflammatory processes and regulation of lymphocyte pools (17–21).

Fatty acids are compounds that can be classified either by chain length or saturation, resulting in the saturated fatty acid (SFA), monounsaturated fatty acid (MUFA), and polyunsaturated fatty acid (PUFA) categories.

PUFA include the essential fatty acids (EFAs) linoleic acid (18:2n6) and α-linolenic acid (18:3n3), which can be only taken with a diet (22). Among PUFA, the most relevant biologic role is played by the omega-3 and the omega-6 series, as they may regulate multiple gene expression and play a fundamental role in cell signaling and inflammation pathways (23, 24). In particular, the pivotal omega-6 arachidonic acid is released from membrane phospholipids when cells are under stress, and becomes the precursor of several pro-inflammatory bioactive compounds, like prostaglandins, thromboxanes, leukotrienes, lipoxines, epoxyeicosatrienoic acids, and hydroxyeicosatetranoic acids (21).

Studies of fatty acids during INS have been mainly focused on biochemical changes during the phase of proteinuria (25). While a decrease in palmitic and arachidonic acids and an increase of oleic acid have been described by Aldamiz Echevarria (26), Das found an increase in palmitic acid and a decrease of 18:0, alpha linolenic acid and EPA (27). This discrepancy may be explained by the fact that Das studied the effect of PUFA n3 supplementation on fatty acid levels, while Aldamiz Echevarria merely described the changes in fatty acid profile. Interestingly, for both authors the pivotal point was the change in the ratio between pro- and anti-oxidant PUFA metabolites, driving to a pro-inflammatory state. However, a pathogenetic meaning cannot be reasonably attributable to these observational findings.

If the changes in fatty acids levels during proteinuria are well-established, to the best of our knowledge no information is available about fatty acids profile in patients with INS on stable remission, although it is well-known that INS is characterized by periods of remission alternating with relapses of proteinuria, mainly in the course of infections, and that fatty acids are strictly involved in the inflammatory processes and in lymphocyte regulation.

The aim of this study is therefore to measure fatty acids levels in patients with INS on stable remission, comparing them to those of a group of matched healthy controls.

## Patients and Methods

The subjects participating in the study were enrolled according to the following criteria:

Patient group: (1) Pediatric patients with a previous diagnosis of idiopathic nephrotic syndrome, based on the following criteria: ratio of proteinuria to creatininuria UPr/UCr >2 mg/mg, and serum albumin <2.5 g/dl. (2) Steroid dependent or frequently relapsing course, according to the definition of the ISKDC: steroid dependent nephrotic syndrome is defined as steroid-sensitive NS with 2 or more consecutive relapses during tapering or within 14 days of stopping steroids; frequently relapsing NS is defined with 2 or more relapses within 6 months, or 4 or more relapses within a 12-months period. (3) Stable remission if UPr/UCr was <0.2 mg/mg, total serum protein >6.4 gr/dl, serum albumin >4 gr/dl (28), and duration of remission more than 30 days from the end of the nearest proteinuric event.

Control group: healthy controls matched for age and gender with neither concomitant inflammatory disease, nor a metabolic or genetic disease, enrolled from a group of children who performed a blood test in preparation to minor urologic corrective surgery.

Body Mass Index of both patients' and controls' groups was calculated, to exclude overweight or obese subjects from the study.

Patients' blood samples were collected for fatty acid profile, serum total protein, albumin, total cholesterol, and triglycerides during scheduled routine visits and obtained after parental consensus and following Helsinki declaration. Blood samples from controls were obtained after parental consensus. The related study protocol was approved by our local Ethical Committee.

Urine was collected from the first morning sample for urine protein and creatinine measurement and urine protein to creatinine ratio (mg/mg) was calculated.

A 3-days dietary diary was collected in a random sample of 10 patients and 10 controls to assess differences in dietary fatty acid intake.

Blood samples were collected on Whatman 903 collection cards BHT (Sigma-Aldrich) pre-treated and stored at temperature of −20°C. Cards were cut and transferred into vials (one vial for each sample) for methylation as described by Marangoni et al. (29). Afterwards, 2 ml of KCl solution (Sigma Aldrich) and 330 μl hexane (Sigma-Aldrich) were added. Samples were first vortexed and then centrifuged 3,000 rpm for 10 min. Finally, hexane layer (the upper layer) was collected from each vial and transferred into gas chromatography vial for fatty acids profile evaluation with fast gas-chromatographer Master GC fast (Dani), equipped with a 15 m fused silica capillary column Omegawax™ 100 (Supelco). The gas chromatography results were analyzed using Clarity software (Data Apex).

Fatty acids were evaluated as percent on the total of fatty acids; the obtained values were used for statistical analysis. Data are provided by clustering fatty acids on the basis of their saturation state (PUFA, MUFA, SFA) and considering among PUFA the omega-3 and omega-6 series.

The activity of the enzymes involved in FA synthesis pathway was evaluated by the product/precursor ratio (30).

Statistical analysis: *t*-test (SPSS 21.0 IBM) was conducted to assess differences in fatty acid profile between patients and controls after a normality test; a *p*-value ≤ 0.05 was considered significant.

In the absence of published data, the power of the test to calculate cohort sample size was not performed.

## Results

**Figure 1:**
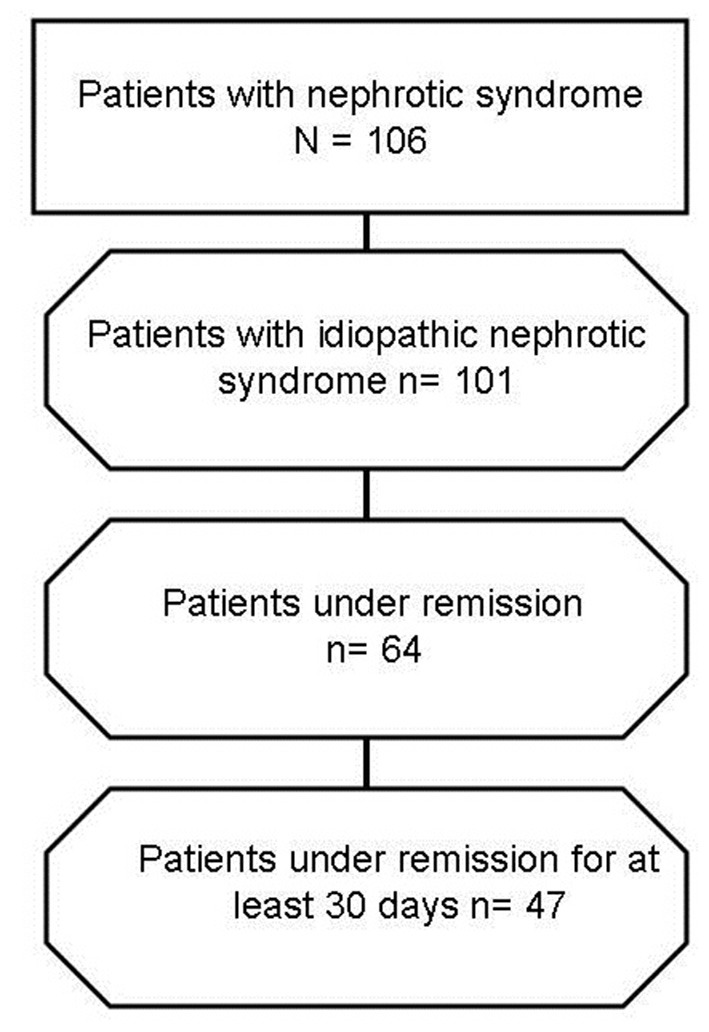
Patients selection flow.

Forty seven pediatric patients, aged 8 ± 4 years, 51% males, with steroid dependent or frequently relapsing nephrotic syndrome were enrolled into the study in the period 01/01/2016–31/12/2019 (Figure 1) and had a blood sampling for fatty acid profile during stable remission (median time elapsed from the nearest occurrence of proteinuria of 60 days, with a range of 32–150 days). Their serum total protein in remission was 7 ± 0.34 g/dl, serum albumin 4.7 ± 0.31 g/dl, and UPr/Cr 0.14 ± 0.04 mg/mg. Thirty-two out of 47 patients were in immunosuppressive therapy, 11 with cyclosporine A, 3 with tacrolimus, and 18 with mycofenolate mofetil.

Sixty healthy children matched for age and gender (age 8 ± 5 years, 51% males) were enrolled as a control group.

None of the patients and controls was overweight or obese. The two groups did not show any statistically significant difference in fatty acid intake at the analysis of the 3-days dietary diaries, as shown in Table 1, and their cholesterol and triglycerides levels were in the normal range.

**Table 1:** Fatty acids dietary intake (g/day) in a random sample of patients and healthy controls.

**Fatty acids dietary intake**		
	**Patients (*****n*** **=** **10)**	**Controls (*****n*** **=** **10)**
SFA	5.851	7.23
18:1n9	5.896	4.74
16:0	2.366	3.12
18:2 n-6	1.478	1.10
18:3 n-3	0.198	0.23
20:4 n-6	0.068	0.063
20:5 n-3	0.040	0.043
22:6 n-3	0.058	0.079

*Values are expressed in g/day; dietary intake was calculated on the basis of a 3-days diary record*.

*n.s., not significant p-value at T-test*.

**Figure 2:**
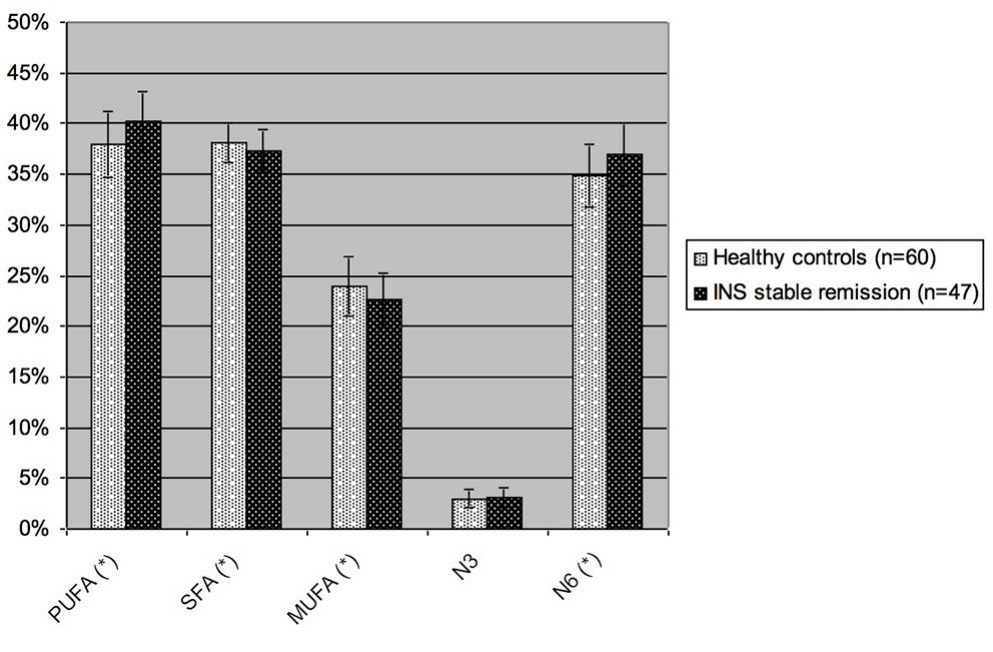
Blood levels of the different groups of fatty acids in children with INS on stable remission and in healthy controls. PUFA, polyunsaturated fatty acids; SFA, saturated fatty acids; MUFA, monounsaturated fatty acids; N3, omega-3 fatty acids; N6, omega-6 fatty acids. Values are expressed as percent of total blood fatty acids. ^*^p-value <0.05, statistically significant at *T*-test.

Considering the main groups of fatty acids (Figure 2), patients with INS on stable remission had significantly higher levels of PUFA and omega-6 than controls (40.17 vs. 37.91% *p*-value 0.0004 and 36.95 vs. 34.79%, *p*-value 0.0005), with consequent lower levels of SFA (37.22 vs. 38.15%, *p*-value 0.02) and MUFA (22.57 vs. 23.94%, *p*-value 0.01). Omega-3 levels were not significantly different in the two groups (3.12 vs. 3.01%, *p*-value 0.53).

**Figure 3:**
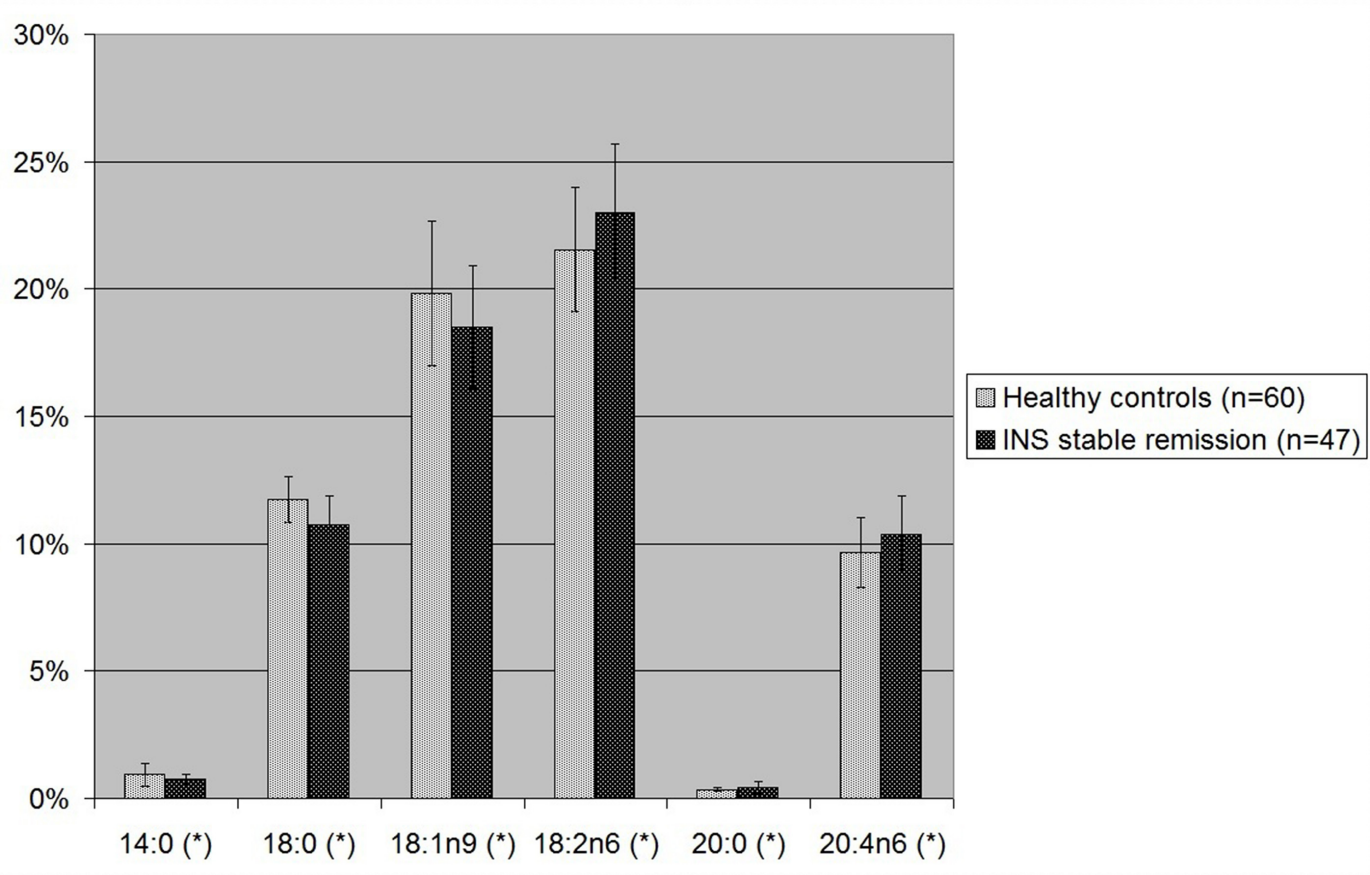
Statistically significant single fatty acids blood levels in children with INS on stable remission and in healthy controls. 18:1n9, oleic acid; 18:2n6, linoleic acid; 20:4n6, arachidonic acid. ^*^p-value <0.05 is statistically significant at *T*-test.

Considering the single fatty acids (Figure 3 and Table 2), levels of omega-6 18:2n6 linoleic acid and omega-6 20:4n6 arachidonic acid were significantly higher in patients with INS during the phase of stable remission than in controls (23.01 vs. 21.55%, *p*-value 0.003 and 10.37 vs. 9.65%, *p*-value 0.01). Moreover, patients with INS, compared to healthy controls, showed significantly lower levels of SFA 14:0 (0.74 vs. 0.92%, *p*-value 0.008) and 18:0 (10.74 vs. 11.74%, *p*-value 0.00002) and MUFA 18:1n9 oleic acid (18.50 vs. 19.83%, *p*-value 0.011).

**Table 2:** Blood levels of single fatty acids and different groups of fatty acids.

	**Healthy controls**		**INS stable remission**	
**Fatty acid**	**Average**	**s.d**.	**Average**	**s.d**.
14:0 (%)	0.92	0.43	0.74	0.23
16:0 (%)	22.61	1.36	22.94	1.37
16:1n7 (%)	0.92	0.29	1.01	0.31
18:0 (%)	11.74	0.92	10.74	1.12
18:1n9 (%)	19.83	2.85	18.50	2.40
18:1n7 (%)	1.26	0.16	1.25	0.21
18:2n6 (%)	21.55	2.43	23.01	2.69
18:3n6 (%)	0.27	0.13	0.30	0.12
18:3n3 (%)	0.22	0.09	0.22	0.06
20:0 (%)	0.34	0.07	0.42	0.23
20:3n9 (%)	0.12	0.04	0.11	0.05
20:3n6 (%)	1.59	0.28	1.58	0.39
20:4n6 (%)	9.65	1.37	10.37	1.52
20:5n3 (%)	0.28	0.16	0.32	0.21
22:0 (%)	1.03	0.20	1.05	0.24
22:4n6 (%)	1.22	0.31	1.14	0.41
22:5n6 (%)	0.50	0.25	0.62	0.38
22:5n3 (%)	0.60	0.14	0.62	0.15
24:0 (%)	1.52	0.29	1.47	0.43
22:6n3 (%)	1.91	0.63	1.90	0.70
24:1n9 (%)	1.68	0.33	1.55	0.44
PUFA	37.91	3.30	40.17	2.97
SFA	38.15	1.93	37.22	2.12
MUFA	23.94	2.88	22.57	2.63
Omega-3	3.01	0.84	3.12	1.03
Omega-6	34.79	3.11	36.95	3.08

*Fatty acid levels are expressed as percent of total fatty acids. P-value calculated at T-test*.

**Figure 4:**
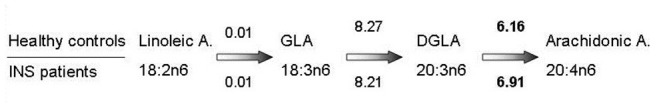
Estimated enzymatic activity through omega-6 pathway. Estimated enzymatic activity from 18:2n6 linoleic acid (precursor) to 20:4n6 arachidonic acid (product). Numbers on the upper side of the figure indicate the estimated activity, calculated as the ratio of product to precursor, in healthy controls, while numbers on the lower side indicate the estimated activity in patients with INS under stable remission. Statistically significant data (*p*-value 0.03) are in bold. GLA, gamma linoleic acid; DGLA, dihomo gamma linoleic acid.

Finally, a significant increase in estimated enzymatic activity in the arachidonic acid synthesis pathway was found in the last step of the omega 6 pathway (Figure 4).

## Discussion

While previous reports have only been focused on the level of fatty acids in patients with proteinuria (26, 27), to the best of our knowledge this is the first study assessing FA profile in children with INS in stable remission. In contrast to the contradictory data on the fatty acids profile available in the literature (26, 27), we were able to demonstrate, in a population of 47 patients, a higher blood level of linoleic and arachidonic acid, and consequently of omega-6 and PUFA, compared to controls.

Immune system derangements resulting in an inflammatory state have been described during proteinuria in nephrotic syndrome and include a few mediators of inflammation, like IL17 A, TNF-α, IFN-γ, TLR-3, and CRP (11–14). Notably, for some of them, the expression was independent from the degree of proteinuria.

Our findings of high omega-6 levels, in particular arachidonic and linoleic acid, are on the same line and can be considered the expression of an inflammatory state, as it involves arachidonic acid (21). Indeed, omega-6 arachidonic acid is the precursor of several pro-inflammatory bioactive compounds, like eicosanoids (21).

However, such findings refer to patients in stable remission, not to patients with proteinuria, and this is the novelty of our study. A high blood level of arachidonic acid, and more generally omega-6 compounds, may be considered a biomarker of a persistent pro-inflammatory state in children with INS even during remission, possibly acting as co-factor involved in the relapse of proteinuria. Availability of larger pool of arachidonic acid, upon a trigger (for instance, an infection) could further contribute to activation of the omega 6 turnover, leading to increased synthesis of pro-inflammatory eicosanoids (the major end-products of arachidonic acid), possibly associated with relapsing proteinuria.

In our patients in stable remission, the high levels of arachidonic acid may be explained by an increase of the estimated enzymatic activity possibly supported by the described higher availability of linoleic acid, observed in the patients, but not in controls, in the last step of the omega 6 pathway, from DGLA to arachidonic acid.

The increased levels of arachidonic acid were associated with increased levels of linoleic acid, the arachidonic acid precursor. The increase in arachidonic and linoleic acid levels cannot be simply explained by a higher dietary intake of omega-6 in children with INS than in controls, as our 3-days dietary diary did not show any statistical difference between the two groups. On the contrary, the origin of the delta in linoleic acid levels between patients and controls could be explained in the first case by a different release from endogenous pools of fatty acids, as part of the general picture of non-communicable chronic diseases with inflammatory expression, in the second case by a reduced activity of metabolizing enzymes of linoleic acid LOX and COX in the patient's group (21). Furthermore, the fatty acids changes that we described did not depend on the use of immunosuppressive agents, as fatty acids levels are not affected by this group of medications (26).

Finally, the existing inverse correlation between blood levels of oleic acid and arachidonic acid (31) may account for the lower levels of MUFA oleic acid in patients than in controls. This association may be mechanistically explained by the biologic balance within families of unsaturated fatty acids (32, 33).

Considering the fatty acid profile described in the literature during the proteinuric phase (26, 27), and the data of this study relating to the remission phase, we can hypothesize that the imbalance of the PUFA n-3/PUFA n-6 ratio reported in the course of proteinuria is a phenomenon that persists even in the course of remission.

Strong points of this work are the homogenous and well-characterized cohort of patients and controls and the novelty of information about a so far scarcely investigated aspect of INS, with possible implications for the management of the disease.

A few limitations should also be recognized. First, the lack of direct measurement either of fatty acid metabolites or of the real enzymatic activity, possibly confirming our explanation of the mechanisms underlying fatty acids profile in patients with INS, otherwise based on the estimated product to precursor ratio. Second, the fact that the dietary record, as regards fatty acids intake, was performed only in 10 INS patients and 10 healthy controls, but the risk of potential inequality in fatty acids dietary intake in the two groups is low, considering that patients with INS on stable remission had no diet limitation and they came from the same geographical area as that of healthy, matched controls. The third limit is the absence of patients' fatty acids profile during relapse, that could help in better evaluating the fatty acids changes during remission, but this would imply longitudinal data, which were not available in all our patients. Also the absence in our series of infrequent relapser patients on full remission without immunosuppressive agents can be seen as a limit of our study, since their fatty acid profile might be similar to controls and serve as a proof of concept of the role of fatty acids as candidate biomarkers for a risk of relapse in children with INS.

In conclusion, our results suggest that patients with INS in stable remission could not be considered “healed,” if they still show an abnormal fatty acid profile compared to that of healthy subjects. Persistently higher than normal levels of either linoleic or arachidonic acid, could be viewed as candidate biomarker for a state of risk of relapse in children with idiopathic nephrotic syndrome.

## Data Availability Statement

The raw data supporting the conclusions of this article will be made available by the authors, without undue reservation.

## Ethics Statement

Ethical review and approval was not required for the study on human participants in accordance with the local legislation and institutional requirements. Written informed consent to participate in this study was provided by the participants' legal guardian/next of kin.

## Author Contributions

Material preparation, data collection, and analysis were performed by ST and M-LS. CT, ED, and AB selected and screened the subjects cohort. VD analyzed the dietary data. The first draft of the manuscript was written by ST. Manuscript was reviewed and edited by AE, LG, and WM. CA, GMa, and GMo critically revised the article and supervision. All authors contributed to the study conception and design, commented on previous versions of the manuscript, read, and approved the final manuscript.

## Conflict of Interest

The authors declare that the research was conducted in the absence of any commercial or financial relationships that could be construed as a potential conflict of interest.

## Abbreviations

PUFA: polyunsaturated fatty acids
MUFA: monounsaturated fatty acids
SFA: saturated fatty acids
EFA: essential fatty acids
DHA: docoexaenoic acid
COX: cyclooxygenase
LOX: lipid oxygenase.
